# Supplementary material for: A random priming amplification method for whole genome sequencing of SARS-CoV-2 virus
Source: BMC Genomics. 2022 May 30;23:406. doi: 10.1186/s12864-022-08563-z (PMC9148844; doi:10.1186/s12864-022-08563-z)
Supplement: Supplementary file 1 — Additional file 1: Supplementary Figure 1. The average depth of coverageobtained after SARS-CoV-2 sequencing using the four different random primingmethods coupled with next-generation sequencing. The virus was 10-fold seriallydiluted (from 2.3 x10^6 pfu/mL, mark as “0” on x axis). Supplementary Figure 2. Read distributions aligning to SARS-CoV-2viral genome following Sequence-Independent, Single-Primer Amplification(SISPA) coupled with Miseq Ilumina sequencing. SARS-CoV-2 virus was 10-foldserially diluted, starting from viral load of 2.3 x 10^6^ pfu/mL (S0),2.3 x 105 pfu/mL (S1), 2.3 x 10^4^ pfu/mL (S2), etc. Numberof reads at each genome position is plotted. [file 12864_2022_8563_MOESM1_ESM.docx]

**Supplementary Figures**

**
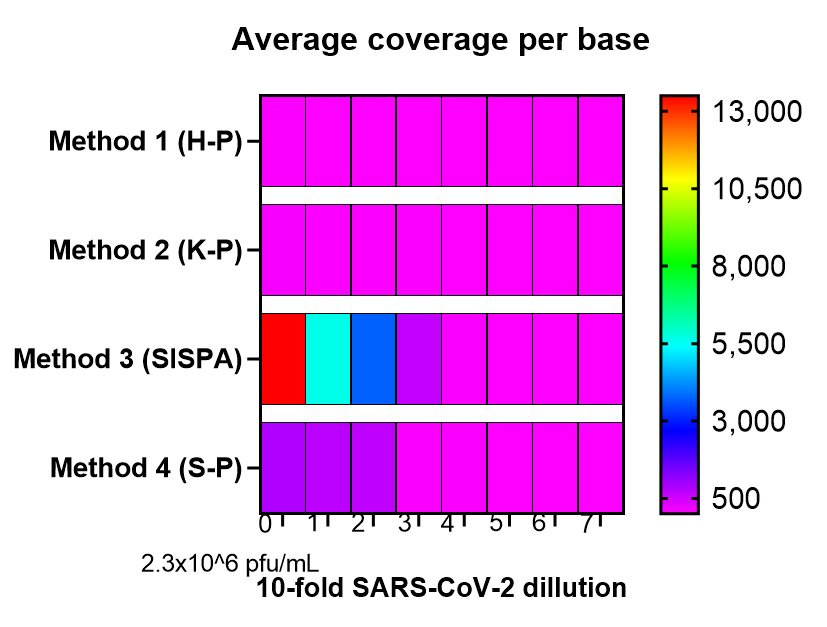
**

Suppl. Figure 1. The average depth of coverage obtained after SARS-CoV-2 sequencing using the four different random priming methods coupled with next-generation sequencing. The virus was 10-fold serially diluted (from 2.3 x10^6 pfu/mL, mark as “0” on x axis).

Suppl. Figure 2. Read distributions aligning to SARS-CoV-2 viral genome following Sequence-Independent, Single-Primer Amplification (SISPA) coupled with Miseq Ilumina sequencing. SARS-CoV-2 virus was 10-fold serially diluted, starting from viral load of 2.3 x 10^6^ pfu/mL (S0), 2.3 x 10^5^ pfu/mL (S1), 2.3 x 10^4^ pfu/mL (S2), etc. Number of reads at each genome position is plotted.
