# Supplementary material for: A random priming amplification method for whole genome sequencing of SARS-CoV-2 virus
Source: BMC Genomics. 2022 May 30;23:406. doi: 10.1186/s12864-022-08563-z (PMC9148844; doi:10.1186/s12864-022-08563-z)
Supplement: Supplementary file 2 — Additional file 2: Supplementarytable 1.Length of amplified S-P products quantified using Tapestation 4200 (Agilent). [file 12864_2022_8563_MOESM2_ESM.docx]

**Supplementary table 1.** Length of amplified S-P products quantified using Tapestation 4200 (Agilent).

| **Virus sample** | **Amplification method** | **Average fragment size (bp)** | **Range of fragment sizes (bp)** |
| --- | --- | --- | --- |
| hCov-19/England/02/2020 (ENG-2) | S-P | 35617 | 5812 - 60000 |
| hCov-19/England/02/2020 (ENG-2) | S-P | 20229 | 5547 – 60000 |
| hCov-19/England/02/2020 (ENG-2) | S-P | 25528 | 22304 – 60000 |
| hCov-19/England/02/2020 (ENG-2) | S-P | 22236 | 19616 - 60000 |
| hCov-19/Scotland/EDB1827/2020 (EDB-2) | S-P | 20238 | 5728 – 60000 |
| hCov-19/Scotland/EDB2398/2020 (EDB-8) | S-P | 32337 | 6128 - 60000 |
